# Early Evidence of Cardiotoxicity and Tumor Response in Patients with Sarcomas after High Cumulative Dose Doxorubicin Given as a Continuous Infusion

**DOI:** 10.1155/2017/7495914

**Published:** 2017-09-26

**Authors:** Raymundo A. Quintana, Jose Banchs, Ridhi Gupta, Heather Y. Lin, Sean D. Raj, Anthony Conley, Vinod Ravi, Dejka Araujo, Robert S. Benjamin, Shreyaskumar Patel, Saroj Vadhan-Raj, Neeta Somaiah

**Affiliations:** ^1^Department of Cardiology, University of Texas MD Anderson Cancer Center, 1515 Holcombe Blvd., Unit 1451, Houston, TX 77030, USA; ^2^Division of Hematology/Oncology, Department of Medicine, Medical University of South Carolina, Charleston, SC 29425, USA; ^3^Department of Sarcoma Medical Oncology, University of Texas MD Anderson Cancer Center, 1515 Holcombe Boulevard, Unit 450, Houston, TX 77030, USA

## Abstract

**Background:**

Despite the dose-dependent response rate of sarcomas to doxorubicin, clinicians limit its cumulative dose due to cardiotoxicity. This study evaluates early evidence of cardiotoxicity in patients treated with high-dose doxorubicin given as a continuous infusion.

**Methods:**

Data was collected on patients who received 90 mg/m^2^ doxorubicin as a continuous infusion and 10 gm/m^2^ ifosfamide for up to 6 cycles as part of a phase II study. Cardiotoxicity was assessed with serial echocardiograms or multigated acquisition scans and serum brain natriuretic peptide and troponin levels. Tumor responses were determined by serial radiographic imaging per RECIST.

**Result:**

Out of the 48 patients enrolled, no patient developed heart failure symptoms; however, 4 out of the 38 (10%) patients with serial left ventricular ejection fraction assessments developed subclinical cardiotoxicity (asymptomatic drop in LVEF ≥ 10%). Twenty-three patients received all six 72-hour cycles of doxorubicin with a mean cumulative dose of 540 mg/m^2^. Among these patients, 4% (*n* = 1) developed subclinical cardiotoxicity. In the advanced disease group (*n* = 39), patients with a complete or partial response received a higher mean cumulative dose than those with stable disease (*p* < 0.033).

**Conclusions:**

Doxorubicin cardiotoxicity can be limited by administering doxorubicin as a continuous infusion, allowing higher cumulative dosing to maximize efficacy.

## 1. Introduction

Sarcomas account for 1% of all malignancies in adults worldwide. These rare tumors originate from mesenchymal cells in different areas of the body. Since the 1970s, doxorubicin (an anthracycline) has been shown to prolong survival in patients with advanced sarcomas [1, 2]. The response to doxorubicin is dose-dependent, with doses above 60 mg/m^2^ considered to be effective in sarcomas [3]. However, the most important limiting factor in the use of doxorubicin is the adverse effect of cardiotoxicity [4]. Anthracycline-induced cardiotoxicity is currently defined as the development of signs or symptoms consistent with heart failure or an asymptomatic decrease in baseline left ventricular ejection fraction (LVEF) ≥ 10% to a level < 50% [5–7]. Prior studies have shown that using a higher cumulative anthracycline dose when administered as a bolus infusion increases the risk of cardiotoxicity in children and adults [8, 9]. In one of these studies, 36% of patients (*n* = 10) developed a ≥10% decline in LVEF and 28% (*n* = 8) of them received a cumulative dose ≥ 400 mg/m^2^ [10]. Therefore, most clinicians limit the cumulative dose to 400–450 mg/m^2^, considered a lifetime maximum in some institutions, to prevent cardiotoxicity regardless of continued therapeutic response. However, it is well documented in studies that cardiotoxicity can be reduced significantly by administration of dexrazoxane, a cardioprotective agent, prior to bolus infusion or by continuous infusion of anthracycline, since anthracycline-induced cardiotoxicity is a peak-dose effect [11–16]. More recently, studies have shown that a brain natriuretic peptide (BNP) level > 100 ng/dl and a troponin I level ≥ 0.08 ng/ml are good predictors of anthracycline-induced cardiotoxicity [17–23]. Herein we report the results of the phase II clinical trial DM00-435, where early doxorubicin-induced cardiotoxicity was assessed by measuring serial LVEF and levels of troponin I and BNP in sarcoma patients receiving high-dose doxorubicin as a continuous infusion and ifosfamide for up to 6 cycles.

## 2. Materials and Methods

From August 2001 to October 2002, forty-eight adult patients enrolled in the phase II clinical trial DM00-435 at MD Anderson Cancer Center who had newly diagnosed high-grade sarcomas, with either advanced disease (American Joint Committee on Cancer Staging system stages IIC, III, or IV) or localized disease with high risk of relapse, were evaluated. All patients received front-line chemotherapy with doxorubicin 90 mg/m^2^ and ifosfamide 10 g/m^2^ every 3 weeks for up to 6 cycles. Doxorubicin was given as a continuous infusion over 72 hours (30 mg/m^2^ over 24 hours × 3 days). Patients were excluded from the study if they had (1) leiomyosarcoma of gastrointestinal origin; (2) structural heart disease or cardiomyopathy; (3) prior radiation therapy; or (4) prior chemotherapy.

### 2.1. Systolic Function Determination

Every patient in our study underwent either a transthoracic echocardiogram (TTE) or a multigated acquisition (MUGA) scan at baseline, midstudy, and end of study. All transthoracic echocardiograms were obtained using the same Vivid 7 or E9 [General Electrics (GE) Healthcare, Milwaukee, WI] ultrasound machine and interpreted by a single reader. Patient's LVEF was calculated from the apical four- and two-chamber views using the biplane method of disks (modified Simpson biplane method). MUGA scans were obtained using the GE Infinia camera with low-energy high-resolution collimator in 180 degrees' configuration (H mode). The GE R-wave trigger was used to gate the acquisition. Patient's red blood cells were labeled with technetium-99m (Ultratag® RBC kit). The LVEF was calculated with data collected from three standard views: anterior, best septal left anterior oblique (45 degrees), and left lateral (70–90 degrees). Some patients underwent additional TTEs and MUGA scans during the follow-up period as part of their future treatment protocols.

### 2.2. Cardiotoxicity

In our study, cardiotoxicity was defined as the development of signs or symptoms consistent with heart failure or an asymptomatic decrease in LVEF ≥ 10% from baseline to a level less than 50% by either TTE or MUGA scan.

### 2.3. Biomarkers

Serum BNP and troponin I measurements were performed at baseline, midstudy, and end of study in all patients. A commercial, multibiomarker panel (Biosite Triage Profiler, Biosite Incorporated®, 9975 Summers Ridge Road, San Diego, CA 92121, USA) was used to quantify BNP and troponin I levels.

### 2.4. Tumor Response Assessment

In patients with advanced disease, we assessed tumor response by computed tomography or magnetic resonance imaging every two chemotherapy cycles according to the Response Evaluation Criteria In Solid Tumors (RECIST) [24, 25]. Treatment responses were classified as follows: (1) complete response (CR): disappearance of all target lesions; (2) partial response (PR): 30% decrease in the sum of the longest diameter of all target lesions; (3) disease progression (PD): 20% increase or more in the sum of the longest diameter of all target lesions; or (4) stable disease (SD): changes in the size of target lesions that do not meet above criteria.

### 2.5. Statistical Analysis

Descriptive statistics such as frequency distribution, mean (±standard deviation), and median (range) were used to summarize patients demographic, tumor characteristic, and cardiac function parameters including LVEF, troponin I, and BNP. Spearman correlation coefficient was used to assess the correlation between the changes of ejection fraction or BNP from baseline to midstudy or end of study with doxorubicin cumulative dose. Chi-square test or Fisher's exact test was used to test differences in categorical variables. Wilcoxon rank-sum test or Kruskal-Wallis test was used to detect differences in continuous variables between groups.

## 3. Results

A total of 48 patients with diverse sarcoma histologies were evaluated for doxorubicin-induced cardiotoxicity and tumor response. Male-to-female ratio was 1 : 1 with a median age of 38.9 years. The clinical features of the studied patients are listed in Table 1. Left ventricular ejection fraction assessments at both baseline and end of study were available for only 38 patients (79%). Twenty-three patients (48%) received six full 72-hour cycles of doxorubicin with a mean cumulative dose of approximately 540 mg/m^2^, and all of these patients had LVEF assessments at baseline and end of study. Midstudy LVEF assessments when done did not differ from end-of-study LVEF and were not always done at the same point in time. The mean number of cycles received among the 48 patients was 4.85 (mean cumulative dose of 433.85 mg/m^2^ and median cumulative dose of 502 mg/m^2^). Fifteen patients (31%) had their infusion duration reduced from 72 to 48 hours due to development of mucositis. Thirty-nine patients (81%) had advanced disease and were evaluable for tumor response. The remaining nine patients (19%) had early disease with high risk of relapse and received doxorubicin as an adjuvant therapy; thus, their response was not evaluable.

None of the patients experienced any clinical signs of cardiotoxicity during the study or during the available follow-up period of 3.7 years (range, 1 month–10.4 years). All pre- and postchemotherapy LVEF measurements were within normal limits (LVEF ≥ 50%). Out of the 38 patients with LVEF assessments at baseline and end of study, only 4 (10%) developed subclinical cardiotoxicity, that is, a drop in LVEF of ≥10% (range, 10–15%). Their clinical characteristics are summarized in Table 2. Interestingly, these 4 patients received a lower mean cumulative dose of doxorubicin than those who did not experience a drop in their LVEF (*p* = 0.04, Table 3). The follow-up period for these 4 patients was 1 year (2 patients) and 4 years (2 patients), during which no clinical heart failure symptoms were noted. All 23 patients that received 6 cycles of doxorubicin over 72 hours were among those evaluated with LVEF assessments at baseline and end of study. Among them, only one patient (4%) developed subclinical cardiotoxicity. Troponin and BNP levels were available in 44 patients at baseline, 48 at midstudy, and 29 at end of study. None of the patients had serum troponin levels ≥ 0.05 ng/ml or BNP levels ≥ 100 pg/ml. Analysis of baseline to end-of-study BNP values showed that none of the patients had a BNP increase > 10 ng/dl. Three out of the four patients who experienced a drop in their LVEF ≥ 10% demonstrated a BNP increase ≤ 10 ng/dl. Nonetheless, the difference was not statistically significant (*p* = 1.0). No statistically significant correlation was found between changes from baseline to end-of-study LVEF (Spearman correlation coefficient, Rho = 0.23, *p*-value = 0.16) or BNP levels (Rho = −0.23, *p*-value = 0.25) and cumulative doxorubicin dose.

Out of the 39 patients (81%) evaluable for tumor response, a complete response was observed in 1 patient (3%), a partial response was seen in 20 patients (51%), stable disease was found in 16 patients (41%), and disease progression was seen in 2 patients (5%). Patients with a complete or partial response received a higher mean cumulative dose than those with stable disease (*p* < 0.033, Table 4).

## 4. Discussion

Cardiotoxicity is a well-known side effect related to the peak dose of doxorubicin. Standard dosing is usually 75 mg/m^2^ per cycle routinely maxed at 6 cycles irrespective of clinical benefit (widely accepted lifetime cumulative doxorubicin dose of 450 mg/m^2^). Continuous infusion (over 72 hours) and dexrazoxane administered prior to bolus infusion are well-known cardioprotective mechanisms employed to mitigate this risk. Our study shows that patients receiving a continuous infusion of high-dose doxorubicin (90 mg/m^2^ per cycle for up to a total of 6 cycles) with a median cumulative dose around 500 mg/m^2^ (majority getting a total of 540 mg/m^2^) did not experience any significant early evidence of cardiotoxicity (based on LVEF, BNP, and troponin I assessment) or clinical heart failure during the available follow-up period of 3.7 years (range, 1 month to 10.4 years). This is in contrast to bolus administration; in a 28-patient study using bolus infusion led to cardiotoxicity in 7%, 21%, and 36% of patients after 200 mg/m^2^, 400 mg/m^2^, and 500 mg/m^2^ of doxorubicin, respectively [10]. In the case of cardiotoxicity during treatment, discontinuation of doxorubicin usually results in improvement of LVEF over time. Echocardiogram monitoring is recommended in all patients at baseline and after 4–6 cycles in high-risk patients, to detect asymptomatic contractile dysfunction that may progress to symptomatic disease with continued therapy [10, 26]. In our study, in the 29 patients who had end-of-study troponin I or BNP levels (including 3 of the 4 patients who had a subclinical decline in LVEF), no significant changes were noted. Although different studies have reported a strong association between BNP levels > 100 ng/dl or a troponin I level ≥ 0.08 ng/ml and early anthracycline-induced cardiotoxicity [17–23], recent evidence suggests that newer assays such as ultrasensitive troponin I or N-Terminal Pro-BNP are more sensitive and should be incorporated in any future studies [24, 25].

While therapy discontinuation may reverse acute systolic dysfunction, there is data to suggest that early evidence of cardiotoxicity correlates with the development of future LVEF impairment; therefore, long-term cardiac follow-up should be considered on an individual basis in these patients.

In our study, patients with CR/PR received higher cumulative dose of doxorubicin. This could be because those with progression stopped therapy sooner, but since only two patients had PD, it suggests that patients who managed to get all cycles of therapy had a higher likelihood of CR/PR. We also found that the 4 patients with subclinical cardiotoxicity received a significant lower mean cumulative dose of doxorubicin. This was probably a result of earlier discontinuation due to unacceptable toxicities or earlier discontinuation upon noting the lower LVEF on midstudy assessment.

Multiple alternate approaches have been evaluated to avoid anthracycline-induced cardiotoxicity, such as structural modifications (epirubicin and mitoxantrone) and liposomal encapsulation of the anthracycline molecule but these have not been able to match the established efficacy of doxorubicin as the standard backbone for sarcoma regimens [27–31]. Currently aldoxorubicin, a tumor-targeted doxorubicin conjugate (doxorubicin attached to an acid sensitive liker and N-*ε*-maleimidocaproic acid hydrazide for preferential tumor uptake), is in clinical trial testing and might offer an alternative in the future with lower cardiotoxicity and higher dose administration. For now, using continuous infusion or dexrazoxane prior to bolus infusion of doxorubicin can allow clinicians to cautiously continue doxorubicin (with frequent LVEF monitoring) beyond the cumulative dose of 450 mg/m^2^, in patients with continued benefit and good tolerance. Since this could potentially open up surgical options for previously unresectable disease and there are limited treatment options for most sarcoma patients, cardioprotection is critical with doxorubicin. Concerns have been raised about the possibility of decreased treatment efficacy when using dexrazoxane with bolus doxorubicin, though a head-to-head study comparing the efficacy with continuous infusion doxorubicin has not been conducted in adults.

Although our study supports the cardioprotective effects of doxorubicin administration as a continuous infusion, high cumulative doses of doxorubicin, especially when given ifosfamide, are not completely devoid of other acute toxicities. In our study, almost one-third of patients (31%) developed mucositis, warranting a decrease in doxorubicin infusion duration to 48 hours, and other expected toxicities, which lead to a subsequent lowering of the dose or discontinuation of therapy. While myelosuppression is a peak-dose-dependent toxicity, mucositis is linked to the duration of exposure/infusion. Patients must be assessed after each cycle to make adjustments in the regimen if needed based on the severity and duration of the acute toxicities, with a higher threshold for acute self-limiting toxicities in the setting of curative therapy. In this study, the median age was 38.9 years (range 19–65), whereas in previous studies of standard dose doxorubicin (75 mg/m^2^) and ifosfamide (10 g/m^2^) the median was 45–48 years [32, 33]. Given the potential for high acute toxicity with this combination, younger patients with fewer comorbidities were probably preferentially enrolled and it is important to note that this high-dose regimen would not be recommended for patients above 65 years.

## 5. Conclusion

In patients with aggressive sarcomas susceptible to doxorubicin, cardiotoxicity can be limited by administering doxorubicin as a continuous infusion over 72 hours per cycle, allowing higher cumulative dosing to maximize efficacy. Physicians should tailor treatment strategies based on the goals of therapy (curative versus palliative), baseline cardiac function, and overall risks when deciding the dose and number of cycles of doxorubicin to administer.

## Figures and Tables

**Table 1:** Patient and treatment characteristics.

Features	*n*	Percent
*Gender*		
Female	24	50.00
Male	24	50.00
*Tumor histology*		
Liposarcoma	9	18.75
Synovial sarcoma	9	18.75
Unclassified sarcoma	9	18.75
Leiomyosarcoma	7	14.58
Osteosarcoma	5	10.42
Ewing's sarcoma/primary	4	8.33
neuroectodermal tumor		
Angiosarcoma	1	2.08
Desmoplastic small round cell tumor	1	2.08
Chondrosarcoma	1	2.08
Malignant phyllodes tumor	1	2.08
Rhabdomyosarcoma	1	2.08
*Location of primary tumors*		
Upper limbs	4	8.33
Lower limbs	8	16.67
Nonextremities	36	75.00
*High-grade sarcoma stage*		
Early disease with high risk of relapse	9	18.75
Advanced disease	39	81.25
*Metastatic at diagnosis*		
Yes	14	29.17
No	34	70.83
*LVEF assessments at both baseline*		
*and end of study*		
Yes	38	79.17
No	10	20.83
*Doxorubicin infusion cycles duration*		
72 hours	33	68.75
72/48 hours	15	31.25

**Table 2:** Clinical characteristics of 4 patients who developed subclinical cardiotoxicity.

Age (years)	Mean cumulative dose (mg/m^2^)	Number of chemotherapy cycles	Baseline LVEF (%)	Baseline troponin I levels (ng/ml)	Midstudy troponin I levels (ng/ml)	End-study troponin I (ng/ml)	Baseline BNP (ng/dl)	Midstudy BNP (ng/dl)	End-study BNP (ng/dl)	Tumor response
45	405	6	65	<0.05	<0.05	<0.05	4.9	4.9	11.7	Neoadjuvant
44	360	4	77	<0.05	<0.05	NA	6.6	4.9	NA	Neoadjuvant
32	525	6	66	<0.05	<0.05	<0.05	4.9	4.9	4.9	Stable disease
53	450	5	68	<0.05	<0.05	<0.05	4.9	4.9	4.9	Stable disease

**Table 3:** Associations between mean doxorubicin cumulative dose and changes in LVEF and BNP.

Variables	Level	Cumulative dose				
		*n*	Mean cumulative dose (mg/m^2^)	Max dose (mg/m^2^)	Median cumulative dose (mg/m^2^)	*p* value
LVEF drop from baseline to end of study	<10%	34	490	540	540	0.040
	>10%	4	435	525	428	
BNP increase from baseline to end of study	<10%	21	521	540	540	0.551
	>10%	5	504	540	540	

**Table 4:** Doxorubicin mean cumulative dose and tumor response.

Variable	Tumor response	*n*	Mean cumulative dose (mg/m^2^)	Min dose (mg/m^2^)	Max dose (mg/m^2^)	Median cumulative dose (mg/m^2^)	*p* value
Doxorubicin mean cumulative dose	Complete response	1	540	540	540	540	0.033
	Partial response	20	490	270	540	540	
	Stable disease	16	416	180	540	450	
